# Multiple splenic abscesses in a rather healthy woman: a case report

**DOI:** 10.4076/1757-1626-2-7340

**Published:** 2009-07-23

**Authors:** Aly Saber

**Affiliations:** Department of General Surgery, Port-Fouad General HospitalAl-Obour Street- Port-Fouad, 11361Egypt

## Abstract

**Introduction:**

Abscess of the spleen is a rather clinical rarity with reported mortality rate up to 47%. The timely and widespread use of imaging methods facilitates early diagnosis and guides treatment, thus improving the prognosis. Most of patients were with recognized risk factors including conditions that compromise the immune system, trauma and intravenous drug abuse in addicts. The surgical treatment by splenectomy is usually the first choice of treatment.

**Case presentation:**

A healthy 45-year-old woman presented to the outpatient clinic with fever, 39°C together with persistent upper-left-quadrant abdominal pain. Her past medical history was free from any chronic debilitating diseases or other predisposing factors. Imaging included chest and abdominal X-ray, followed by a CT scan of the upper abdomen.

**Conclusions:**

Splenic abscess is an unusual and potentially life-threatening disease with a diagnostic challenge due to the nonspecific clinical picture and diagnosis confirmed based mostly on imaging studies. Multiple splenic abscesses are very rarely encountered in surgical practice with a reported high mortality rate in neglected and untreated cases. Splenectomy is a safe procedure for patients with splenic abscess.

## Introduction

Abscess of the spleen is a rather clinical rarity as only about 600 cases have been described so far in the international literature [1,2]. Most of patients were with recognized risk factors including conditions that compromise the immune system, such as endocarditis, diabetes mellitus, congenital or acquired immunodeficiency and the administration of immunosuppressive medication [2]. Trauma and intravenous drug abuse in addicts are additional predisposing factors for splenic abscesses [3]. Solitary splenic abscess, however, with lack of any obvious risk factors is very rare [3,4]. The clinical manifestations of splenic abscesses usually include left upper abdominal pain, fever, nausea, vomiting and anorexia may be also present in various combinations [5]. Imaging by ultrasound and computed tomography is suggestive specially when correlated with clinical data [6].

The surgical treatment by splenectomy is usually the first choice of treatment, but image-guided percutaneous drainage is also a therapeutic option in selected cases [7].

## Case presentation

A healthy and otherwise normal 45-year-old woman from the rural middle Delta in Egypt presented to the outpatient clinic with fever, 39°C together with persistent upper-left-quadrant abdominal pain. She was a heavy-duty worker as a farmer, helping her husband nearly all the day time.

Her past medical history was free from any chronic debilitating diseases such as diabetes, liver or renal diseases. Searching for other predisposing factors known to cause splenic abscesses was negative such as metastatic and distant hematogenous infection and a contiguous site of infection. Moreover, the patient recent medical history was free from any surgical operation, recent hospital admission or trauma.

Clinical examination reproduced localized pain and tenderness in the left hypochondrium together with coated tongue and toxic face. The patient radial pulse was 110 beats/minute. Constitutional symptoms as nausea and anorexia were present.

Laboratory testing for hematological and biochemical controls was performed and revealed leukocytosis while other tests were otherwise within the normal ranges.

**Figure 1.**
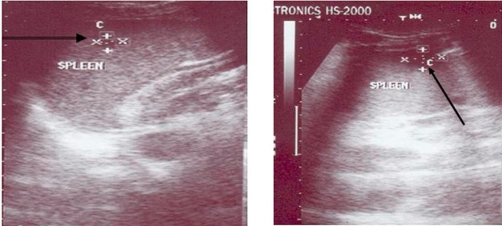
A pre-operative ultrasonographic study showing well-defined splenic focal lesions, cystic in nature and multiple in number.

**Figure 2.**
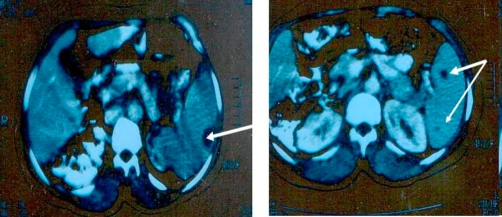
A pre-operative CT scan study showing well-defined focal lesions, cystic in nature and multiple in number located just subcapsular and deep in the splenic parenchyma.

**Figure 3.**
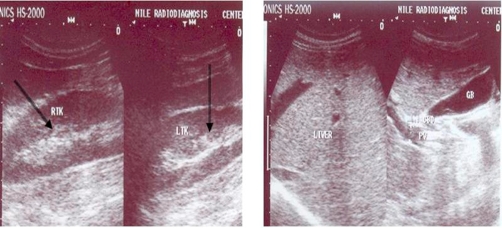
Pre-operative ultrasonographic figures showing normal right and left kidneys as well as normal liver and biliary system.

Imaging study included chest and abdominal X-ray, followed by abdominopelvic ultrasonogam and CT scan of the upper abdomen. Both chest X-ray and CT scan of chest denoted clear lung fields and free costophrenic angles. Both ultrasonography and CT scan showed mild enlarged spleen with multiple well-defined cystic focal lesions; the largest measure 2 × 1.5 cm (Figures 1, 2). Liver, kidneys, pancreas and para-aortic region are normal in ultrasonography and CT scan (Figure 3).

**Figure 4.**
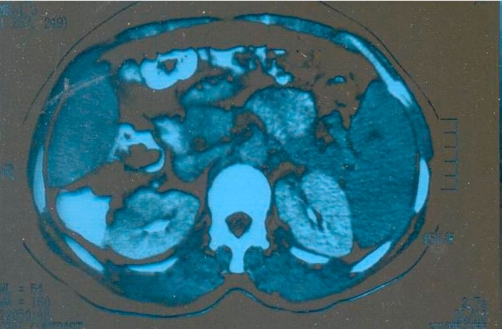
A pre-operative CT scan study showing normal right and left kidneys as well as pancreatic and para-aortic regions.

**Figure 5.**
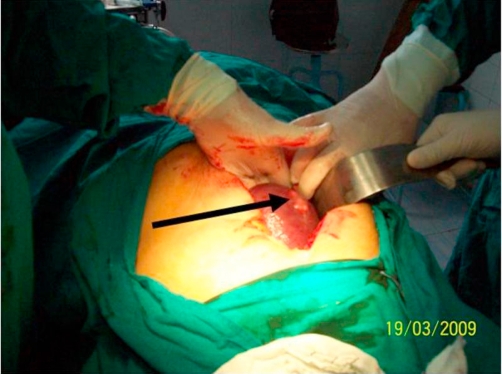
An operative photograph showing the mildly enlarged spleen in situ and one of the splenic abscesses is in view [indicated by the arrow].

**Figure 6.**
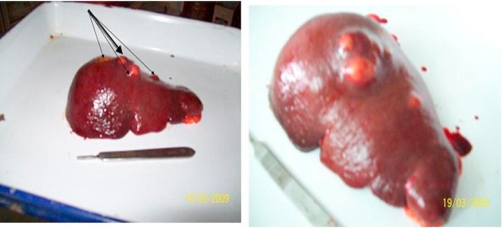
A post-operative photograph showing four abscesses located on the serosal surface of the spleen taken from the side- view and from above.

The patient underwent laparotomy for splenectomy under general anesthesia. Spleen was found mildly enlarged with three small abscesses pointing through the serosal surface. Surgery was completed with smooth operative course and the patient has been found healthy with complete relief from all disease-associated symptoms in the early postoperative period. Operative and post-operative photographic pictures were taken showing the enlarged spleen in situ and the abscesses on splenic surfaces as well as the cavity of the abscesses when laid open (Figures 4, 5, 6).

The histopathological study of the spleen revealed that the cause of these multiple splenic abscesses was nonspecific inflammatory process and the bacteriological study denoted that pathogens detected include staphylococcus and streptococcus.

In the early postoperative period, within 2 weeks, the patient was referred to our internist and hematologist for proper immunization program and antibiotic prophylaxis.

## Discussion

Splenic abscess is a rare entity, with a reported frequency of 0.14-0.7% in autopsy series. Its reported mortality rate is still high, up to 47%, and can potentially reach 100% among patients who do not receive antibiotic treatment. Appropriate management can decrease the mortality to 14% [1,2,8,9].

The timely and widespread use of imaging methods facilitates early diagnosis and guides treatment, thus improving the prognosis. Ultrasound is used as a preliminary diagnostic modality, which is often followed by CT scan [6]. However, ultrasonography can not discriminate between abscess and infarct in some cases, while computed tomography is the examination of choice. Splenic abscesses appear as focal areas of low attenuation with no inflammatory rim [8,10].

In the present case, the author found that abdominal ultrasonography could diagnose the presence of multiple splenic focal cystic lesions and the same finding was confirmed by C T abdominal scan.

The finding of this imaging study was suggestive specially when correlated with clinical data including left upper abdominal pain, fever and other constitutional symptoms [5,6,8].

There is often overlap in the imaging appearance alone, so the clinical setting is very helpful in differential diagnosis. Common symptoms in patients with splenic abscess are fever, abdominal pain and nausea and vomiting [11,12].

Solitary splenic lesions are unusual [13] and multiple splenic lesions whether of infectious or inflammatory origin are also rare [2,3,14]. Multiple splenic abscesses are still extremely rare finding in the clinical practice encountered mostly in immunocompromised patients [15] and in those with underlying malignancies [16].

An interesting recent study published in 2008 stated that abscess of the spleen is a rare discovery and put a hint about this rarity as only 600 cases have been described so far in the international literature [2].

To clarify the rarity of splenic abscess in clinical aspect, the medical records of one institution were retrospectively reviewed and only six cases of splenic abscesses seen within ten years between 1989 and 1999 were identified [5]. Also, reviewing of medical records of patients with a splenic abscess treated in Santiago, Chile within 18 years between 1987 and 2005 revealed only seven patients [7]. A report from Greece, Attikon University Hospital showed that only three cases were mentioned [2]. Another study reported only 67 cases of splenic abscess in a medical center of Taiwan during a period of 19 years [8]. In Pakistan, Karachi, the Aga Khan University Hospital has recorded only 27 patients with splenic abscess within 19 years diagnosed from 1988 to 2007 [6]. A 10-year (1996-2005) retrospective review of case records from a single centre in Singapore of 800-bed general hospital revealed 21 cases with splenic abscess [1].

Most of these patients, if not all, according to these studies and others of same interest, are with recognized risk factors [2,5-8]. These include the synchronous presence of conditions that compromise the immune system, such as endocarditis, diabetes mellitus, congenital or acquired immunodeficiency and the administration of immunosuppressive medication as well as trauma [1,2,3,8].

On the other hand multiple splenic abscesses of unclear aetiology are a very rare finding in clinical practice [17].

The treatment modalities of splenic abscess are antibiotic therapy whether in conjunction with splenectomy, percutaneous drainage or aspiration or antibiotic therapy alone [7,8]. But better outcome was found in patients with splenectomy than patients with percutaneous drainage or aspiration in solitary splenic abscess [2,8].

The patient of this case gained benefit from splenectomy and passed a very smooth postoperative course where the fever declined dramatically and all the constitutional symptoms disappeared earlier within the first 48 hours after surgery.

Although overwhelming postsplenectomy sepsis is a rare event, it is devastating and often lethal [18]. Guidelines for planned splenectomy indicated prophylactic vaccination 15 days before surgery as well as antibiotic prophylaxis [19]. These guidelines include immunizations, antibioprophylaxis, and education. Immunizations against Streptococcus pneumoniae, Neisseria meningitidis, Hamophilus influenzae, and influenza should be administered. Antibioprophylaxis during 2 to 5 years following splenectomy in children, and 2 years in adults is recommended. Furthermore, long-term education is mandatory. Application of preventing measures is effective and patient’s education remains the cornerstone of prevention [20].

## Conclusions

Splenic abscess is an unusual and potentially life-threatening disease with a diagnostic challenge due to the nonspecific clinical picture. Splenic abscess should be suspected in febrile patients with left upper quadrant tenderness and leukocytosis, and diagnosis confirmed based mostly on imaging studies.

Multiple splenic abscesses are very rarely encountered in surgical practice with a reported high mortality rate in neglected and untreated cases. High index of suspicion and liberal use of imaging studies is essential for timely diagnosis. Splenectomy is a safe procedure for patients with splenic abscess.

## Abbreviation

CT: Computing tomography
